# Social Hypersensitivity in Bipolar Disorder: An ERP Study

**DOI:** 10.1192/j.eurpsy.2022.425

**Published:** 2022-09-01

**Authors:** Y. Kwan, J. Lee, S. Hwang, S. Choi

**Affiliations:** 1 Yonsei University Wonju College of Medicine, Psychiatry, Wonju, Republic of Korea; 2 Duksung Women’s University, Clinical Psychology, Soul, Republic of Korea; 3 Yonsei University Wonju College of Medicine, Artificial Intelligence Bigdata Medical Center, Wonju, Republic of Korea

**Keywords:** reward hypersensitivity, social reward, bipolar disorder

## Abstract

**Introduction:**

Bipolar Disorder (BD) is a disorder in which cognitive function is relatively preserved but social functioning is markedly impaired. Interestingly, studies on BD show that the patients have a strong desire for social rewards. Hypersensitivity to social rewards in BD has not yet been sufficiently examined through experimental methods, although recent studies have pointed out that their reward hypersensitivity is the cause of symptoms and dysfunction.

**Objectives:**

The purpose of this study was to investigate whether patients with BD are hypersensitive to social rewards using the social value capture task.

**Methods:**

Groups of 25 BD and healthy control (HC) each completed the social value attention capture task. This task consists of a practice phase in which associative learning of social rewards with specific stimuli occurs, and a test phase in which the stimuli associated with rewards appear as distractors during the participants performing a selective attention task. We also recorded event-related potential (ERP) in the practice phase in order to investigate BDs’ cortical activity for social reward.

**Results:**

showed significantly decreased accuracy rate and increased reaction time in the high social reward-associated distractor trials of the test phase in the BD compared to the HC. As a result of analysis in ERP components, P3 amplitude for social reward was significantly greater in the BD than the HC.

**Conclusions:**

BD patients exhibit behavioral and physiological hypersensitivity to social rewards that might contribute to social dysfunction.

**Disclosure:**

No significant relationships.

